# Epidemic Cholera in Campeache

**Published:** 1834

**Authors:** 


### XIV.—Epidemic Cholera in Campeache.

We have been favored by Dr. K. M'Kinney, lately from Yucatan, with some memoradums on the cholera, as it prevailed in the town of Campeache, in that peninsula. Having published a long and interesting letter from his friend Dr. Perrine, on the same subject, we shall merely give a few extracts from the paper of Dr. M'Kinney.

"Among the natives of Campeache, there is much more nervous irritability than is found in the habitants of higher latitudes."

"July 4th, only two or three attendants, and near fifty patients. The calomel was exhausted yesterday, and none could be procured for the hospital. I ordered blue pill 30 grs. every hour, with infusion of black tea and external stimulants—some little improvement in the results. Some cases, apparently the most hopeless, recovered. I generally ordered for male adults, the most numerous class of my patients, 30 grs. every hour till they had taken three doses, but if the vomiting and purging continued, to give it every hour. As I had found in private practice, that the irritability of the stomach was frequently allayed by giving a spoonful of cold water every five or ten minutes, according to the violence of the thirst, I abandoned the use of warm drinks entirely, gave cold well water and cold rice water every few minutes, but in very small quantity. It allayed thirst better, and provoked vomiting much less than larger draughts, or even small draughts of warm liquids."

"I attended a neighborhood around the bridge of St. Francisco. The persons are principally retailers of liquors and provisions, and butchers. I had 24 patients in this vicinity of all sexes, ages and habits; most of them were attacked with malignant cholera, and some of them in its most violent form. I gave 20 grs. of calomel with ½ gr. of opium every half hour, or every hour, according to the violence of the symptoms, till the vomiting ceased. When the diarrhœa was urgent, I used suppositories of 2 to 3 grs. of opium, which produced the most decidedly beneficial effects. Opium given in this manner is much milder in its effects, and in my opinion more beneficial, than when introduced into the stomach; and were I to treat cholera again, I would introduce no opium into the stomach, except in the form of sulphate or acetate of morphina. External frictions and stimulants were used in all cases—and the only food in the first days of convalescence, was their arrowroot jelly and rice water. Of the 24 thus treated, 20 recovered."

"In the same neighborhood 26 others were attacked, and treated with ether and other stimulants. Of these, 18 died."

"There should not be too great a haste in burying the dead. Those who die of cholera, are not disposed to sudden putrefaction, and consequently cannot injure the living. Many of them while in a state of asphyxia, ceasing to breathe, are to all appearance dead. In Campeache, two or three revived while being carried to the grave yard; and in Minuco, a young woman actually in the grave revived, when they began to fill it up, was taken out, and lived till the next day. A servant, who was to all appearance dead, was stretched in the back yard—while his master was preparing for his burial, a violent shower thoroughly drenched the body—the undertakers came, and to their astonishment and dismay, found the supposed corpse seated, and trying to leave his hard bed.

He was taken into the house and recovered, and is alive at the present day. I doubt not many have been buried alive."

"A few cases were treated with calomel alone, without opium, and the vomiting, cramps, and even purging, appeared to be checked as soon as when opium or its saltz was used. I consider it of some importance to give the calomel floated on water, as it is thereby thrown into the stomach almost dry, and notwithstanding the vomiting, a portion will adhere to the coats of the stomach. When given in pills, it cannot produce its effects so soon as when given in powder, and may be thrown entirely off by vomiting. I have given calomel occasionally in much larger portions, but I could not see that its effects were more speedy, than when given in scruple doses, and repeated, according to the urgency of the symptoms. To calm the irritability of the stomach, nothing appeared to answer so well as a table spoonful of cold water, every five minutes."

